# Lung adenocarcinoma with micropapillary component presenting with metastatic scrotum tumor and cancer-to-cancer metastasis: A case report

**DOI:** 10.1186/1757-1626-1-162

**Published:** 2008-09-19

**Authors:** Kiyoshi Mori, Riko Kitazawa, Takeshi Kondo, Sohei Kitazawa

**Affiliations:** 1 Division of Pathology, Department of Pathology, Kobe University, Graduate School of Medicine, Japan

## Abstract

A 54-year-old man was admitted to the hospital presenting with a 3-month history of sclerosing dermal lesion in the external genitalia. A scrotal skin biopsy revealed a poorly-differentiated adenocarcinoma, immunohistochemically positive for cytokeratin 7 (CK7) and for thyroid transcription factor 1 (TTF-1), and negative for CK20. One month after admission, he died of respiratory failure. At autopsy, a consolidating lesion with vague margin was noted in the left lung as well as a well-circumscribed nodule in the right lobe of the thyroid. Histopathologically, pulmonary lesion was adenocarcinoma with a micropapillary component. On the other hand, thyroid tumor was diagnosed as a follicular variant of papillary carcinoma with foci of micropapillary adenocarcinoma. Positive immunohistochemistry for surfactant protein on micoropapillary component was useful to confirm that micropapillary component was of lung adenocarcinoma origin.

## Background

Carcinomas with micropapillary components have been reported as rare but poorly prognostic tumors in various organs including the breast, urinary bladder, lung, ovary, and major salivary glands [1-4]. Not only the characteristic morphologic pattern but also aggressive manifestations including severe lymph node metastases and lymphovascular infiltration make the tumors as distinctive entity of adenocarcinoma. In this report we describe a case of lung adenocarcinoma with micropapillary component presenting as metastatic skin lesion and cancer-to-cancer metastasis to thyroid papillary carcinoma.

## Case presentation

**Figure 1:**
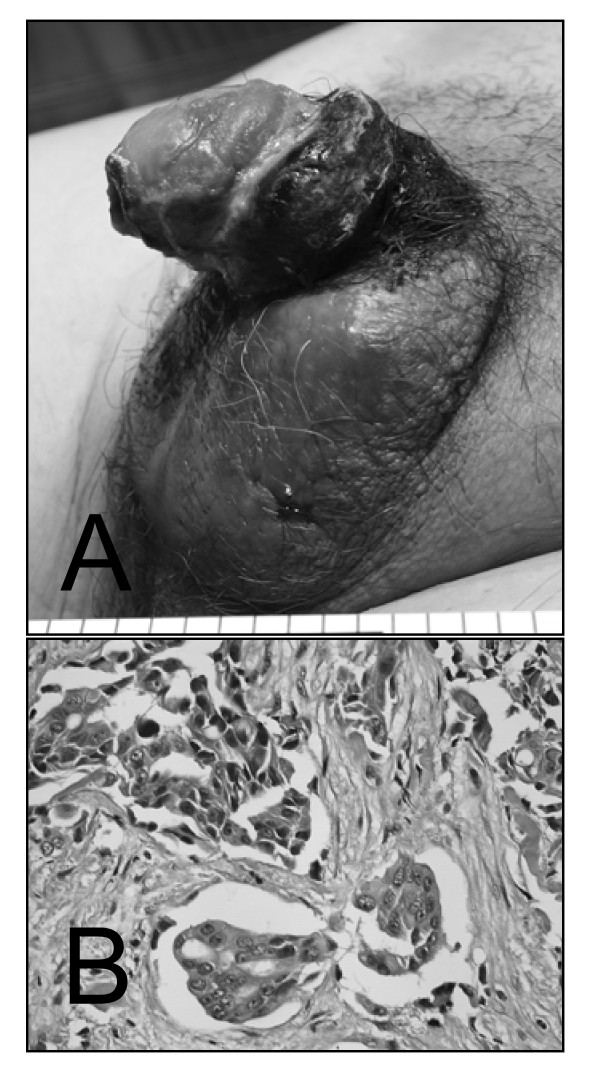
**A sclerosing dermal lesion on the external genitalia is noted at admittance (A).** A core needle biopsy revealed that poorly differentiated adenocarcinoma showing extensive infiltration with frequent lymph vessel invasion (B, H.E. ×400).

A 54-year-old Japanese man was admitted to our hospital complaining of a 3-month history of a sclerosing dermal lesion on the external genitalia (Fig. 1A). Computed tomography (CT) revealed numerous nodules invading various parenchymal organs and lymph nodes, suggesting an aggressive malignancy with systemic metastases. Histologic analysis of core needle biopsy samples from scrotal skin disclosed poorly-differentiated adenocarcinoma infiltrating the dermis with frequent lymph vessel invasion (Fig. 1B). Immunohistologically, the tumor cells were stained positive for cytokeratin 7 (CK7) in the cytoplasm and for thyroid transcription factor 1 (TTF-1) in the nucleus, but negative for cytokeratin 20 (CK20), suggesting that the tumor cells originated from either thyroid cancer or lung adenocarcinoma. The patient died of respiratory failure one month after admission, and an autopsy was conducted to disclose the origin of the cancer.

### Pathologic Findings

**Figure 2:**
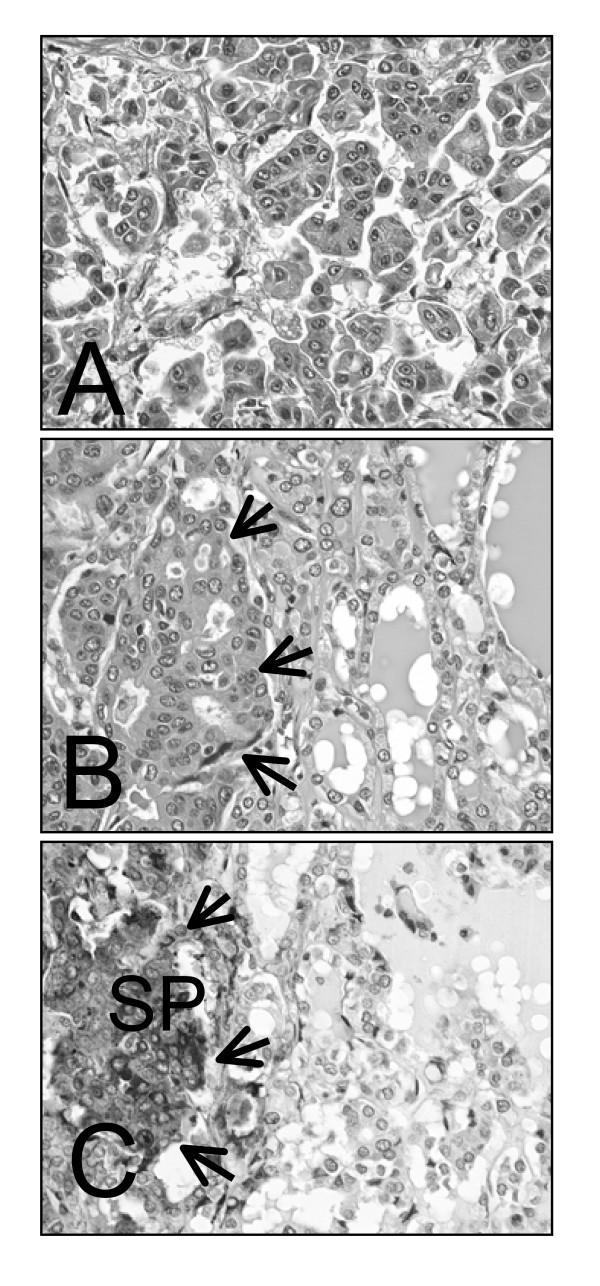
**At autopsy, a massively spreading tumor was noted in the right lung with characteristic histopathological feature of small clusters with no fibrovascular cores surrounded by clear spaces, a typical structure of micropapillary component (A, H.E. ×400).** This MPC was also noted around the thyroid papillary carcinoma (B, arrows, H.E. ×400). Immunohistochemical analysis revealed that surfactant protein (SP) was positive in MPC (C, arrows) but negative in papillary carcinoma (C, IHC, ×400), indicating that MPC was derived from adenocarcinoma of the lung with MPC.

Grossly, massively spreading lesion (approximately 3 × 3 × 3 cm) with vague margin was noted in the right lung accompanying lymphangitic carcinomatosis, as well as a well-circumscribed nodule (8 mm in diameter) in the right lobe of the thyroid. We confirmed aggressive metastatsis at various loci including liver, bilateral adrenals, peritoneum, cardiac muscle of the right atrium, both thoracic (Th5) and lumbar (L5) vertebra, and lymph nodes within trunk except for head and neck. Microscopic examination revealed that the lung tumor was composed of adenocarcinoma presenting characteristic morphological structure of small tumor clusters with no fibrovascular cores surrounded by clear spaces (Fig. 2A), so-called 'carcinoma with micropapillary component (MPC)'. Moreover, these MPCs showed severe lymphovascular infiltration in parenchyma and in pleura. All these metastatic lesions were consisted of MPCs, whereas thyroid tumor was histopathologically composed of mainly follicles with atypical nuclear features such as ground-glass appearance and nuclear grooves characteristic of thyroid follicular variant of papillary carcinoma surrounded by MPCs (Fig. 2B). To further characterize this somewhat complex thyroid nodule with MPC-like clusters, we conducted immunohistochemical analysis.

### Immunohistochemical Findings

Immunohistochemical analysis of tissue samples taken at autopsy confirmed that tumor cells in the lung MPCs, thyroid papillary carcinoma, and thyroid MPC-like clusters were all positive for CK7 but negative for CK20. TTF-1 staining demonstrated weakly positive in lung MPC and in thyroid papillary carcinoma follicular variant, whereas thyroid MPC-like clusters were negative. Furthermore, thyroglobulin was positive in thyroid papillary carcinoma with follicular variant, while negative in the MPC-like clusters in both thyroid and lung. On the other hand, lung adenocarcinoma and metastatic MPCs, including MPC in thyroid tumor (Fig. 2C), were exclusively positive for surfactant protein. These findings suggested that MPC-like clusters in the thyroid papillary carcinoma was metastatic tumor derived from lung cancer. Immunohistochemical analysis for D2-40 confirmed that the MPC was not located within a lymph vessel but in clear tissue space.

## Discussion

Carcinomas with MPC were rare but aggressive tumors with poor prognosis. According to the review article, these tumors have predilections for mammary gland, urinary bladder, lung and salivary gland [1]. Also colon-derived cases are reported recently [5]. Histopathologically, cancer with MPC is characterized by clusters of tumor cells with no fibrovascular cores surrounded by clear spaces resembling dilated lymphatic channels, and inner surface of the cavity demonstrates negative immunoreactivity for endothelial (e.g. factor VIII-related antigen and CD31) nor lymphatic endothelial markers (e.g. D2-40). Additionally each cluster has inversed basal-apical polarity of glandular structure [5,6], suggesting that stroma-facing (basal) surface of the cells acquires apical secretory properties and secrets molecules responsible for stromal/vascular invasion such as metalloproteinases, making this type of tumors aggressive [1].

In this case, because immunohistochemical analysis for TTF-1 and CK7/CK20 suggested that primary focus can either be thyroid cancer or lung adenocarcinoma, we used surfactant protein as a selective marker for the lung cancer. With positive immunohistochemistry for the surfactant protein and systemic analysis by autopsy, we concluded that this case is a primary lung adenocarcinoma with MPC with cancer-to-cancer metastasis. Lack of obvious head and neck lymph node metastasis (e.g. deep cervical nodes) in spite of massive lymph nodes metastases seen in thoracoabdominal cavity also supported that MPC is derived from the lung.

## Conclusion

In summary, we reported an autopsy case of lung cancer with MPC metastasizing to the thyroid papillary carcinoma. Wide spreading systemic metastasis clearly illustrates the aggressive character of cancers with this particular histopathological feature.

## Competing interests

The authors declare that they have no competing interests.

## Authors' contributions

KM performed the autopsy and histological examination, and was a major contributor in writing the manuscript. RK assisted the histological examination. TK assisted immunohistochemical evaluations. SK supported case analysis and writing the manuscript. All authors read and approved the final manuscript."

## Consent

Written informed consent was obtained from the patient' family for publication of this case report and accompanying images. A copy of the written consent is available for review by the Editor-in-Chief of this journal.
